# N-Terminal Fusion Tags for Effective Production of G-Protein-Coupled Receptors in Bacterial Cell-Free Systems 

**Published:** 2012

**Authors:** E.N. Lyukmanova, Z.O. Shenkarev, N.F. Khabibullina, D.S. Kulbatskiy, M.A. Shulepko, L.E. Petrovskaya, A.S. Arseniev, D.A. Dolgikh, M.P. Kirpichnikov

**Affiliations:** Shemyakin and Ovchinnikov Institute of Bioorganic Chemistry, Russian Academy of Sciences, Miklukho-Maklaya Str., 16/10, Moscow, Russia, 117997; Faculty of Biology, Lomonosov Moscow State University, Leninskie Gory 1/12, Moscow, Russia, 119991

**Keywords:** Cell-free expression, GPCR, translation initiation

## Abstract

G-protein-coupled receptors (GPCR) constitute one of the biggest families of membrane proteins. In spite of the fact that they are highly relevant to pharmacy, they have remained poorly explored. One of the main bottlenecks encountered in structural-functional studies of GPCRs is the difficulty to produce sufficient amounts of the proteins. Cell-free systems based on bacterial extracts from*E. coli*cells attract much attention as an effective tool for recombinant production of membrane proteins. GPCR production in bacterial cell-free expression systems is often inefficient because of the problems associated with the low efficiency of the translation initiation process. This problem could be resolved if GPCRs were expressed in the form of hybrid proteins with N-terminal polypeptide fusion tags. In the present work, three new N-terminal fusion tags are proposed for cell-free production of the human β2-adrenergic receptor, human M1 muscarinic acetylcholine receptor, and human somatostatin receptor type 5. It is demonstrated that the application of an N-terminal fragment (6 a.a.) of bacteriorhodopsin from*Exiguobacterium sibiricum*(ESR-tag), N-terminal fragment (16 а.о.) of RNAse A (S-tag), and Mistic protein from*B. subtilis*allows to increase the CF synthesis of the target GPCRs by 5–38 times, resulting in yields of 0.6–3.8 mg from 1 ml of the reaction mixture, which is sufficient for structural-functional studies.

## INTRODUCTION 

Integrated membrane proteins (MPs) participate in a number of processes essential for single-cell and metazoan organisms. These proteins are responsible for cellular energetics, intercellular recognition, signal transduction, and transport of various substances through the cell membrane [1]. Recent data indicate that MPs make up over 25% of all amino acid sequences in the genomes of higher organisms, including the human genome. G-protein-coupled receptors (GPCR) are among the most pharmacologically important MP classes. Over 800 GPCR genes have been identified in the human genome [3], and membrane receptors of this class are the targets of ~30% of modern drugs [4]. GPCRs are characterized by homological spatial organization and contain seven transmembrane (TM) helices, as well as the extracellular N- and intracellular C-terminal regions [5]. The binding sites of low-molecular-weight ligands localize in the TM domain of the receptor, whereas peptide hormones and regulatory proteins interact with the N-terminal region and extracellular loops [5]. 

GPCRs are of particular interest for pharmacological research; however, structural and functional investigations of these receptors are complicated [5] because of the infeasibility of isolating a sufficient amount of the protein from natural sources and the problems concerned with designing high-performance systems to heterologously produce these MPs [6]. Over the last decade, the joint use of expression systems based on eukaryotic cells and new methods of X-ray structure analysis has enabled to determine the spatial structure of a series of GPCRs [5], including the human β2-adrenoreceptor (β2AR) [7] and human muscarinic M2 and M3 cholinoreceptors (mAChR) [8, 9]. These studies have led to a better understanding of the principles of the spatial organization of GPCR. However, a thorough investigation into the functional dynamics and mechanisms of membrane receptor functioning requires the use of high-resolution spectroscopic methods, such as heteronuclear NMR spectroscopy [10]. The current NMR spectroscopy methods require milligram amounts of protein samples labeled with stable isotopes ( ^2^ H, ^13^ C, ^15^ N) [10], which are expensive when eukaryotic systems are used. Meanwhile, the use of conventional bacterial expression systems for GPCR production often does not allow to achieve high yields of the target protein and is complicated due to the necessity to develop re-naturation protocols [11]. 

Cell-free (CF) expression systems [12], and in particular those based on bacterial extracts, have recently gained increasing popularity as an alternative tool for the recombinant production of MPs [13]. As compared with the systems based on cell production, CF systems have a number of advantages, including exclusive production of the target protein, the possibility to synthesize toxic proteins, simple procedure for synthesizing selectively isotope-labeled samples, and the possibility of direct introduction of various agents and cofactors to the reaction mixture to stabilize the native spatial structure of the synthesized protein in the solution [12, 13]. Thus, the components of membrane-mimicking media, such as detergent micelles, lipid/detergent bicelles, liposomes, and lipid–protein nanodiscs, can be added to the reaction mixture to produce soluble MPs [13–15]. 

According to the published data, direct expression of GPCR genes in CF systems is inefficient [14, 16–18]. The low efficiency of the translation initiation process [18], due to the formation of a secondary structure of the 5-prime end mRNA fragment, is among the possible reasons [19, 20]. In most cases, this problem can be solved and the desired level of GPCR production can be attained by inserting additional nucleotide sequences encoding N-terminal polypeptide fusion tags, such as the fragment of the protein 10 leader sequence of bacteriophage T7 (T7-tag, 11 a.a.; hereinafter, the sequence length is given with allowance for the N-terminal Met residue) [14,16], the thioredoxin protein from *E. coli* (TRX) [17], or 1–6 a.a. long synthetic sequences [18] at the 5-prime end of the target protein gene. 

Three novel N-terminal fusion tags are proposed in this work in order to increase the efficiency of cell-free production of human GPCR by the example of β2AR, M1-mAChR, and somatostatin receptor type 5 (SSTR5). It is shown that the use of nucleotide sequences encoding the N-terminal fragment (6 a.a.) of bacteriorhodopsin from Gram-positive bacteria *Exiguobacterium sibiricum* (ESR-tag), the N-terminal fragment (16 a.a.) of ribonuclease A (N-terminal fragment of S-peptide, S-tag), and Mistic protein from *Bacillus subtilis* allows to increase the receptor yield by 5–38 times, providing a sufficient level of target protein production for further structural and functional studies. 

## EXPERIMENTAL 

**Design and cloning of the **

*GPCR* **genes with additional 5-prime end sequences**

**Fig. 1:**
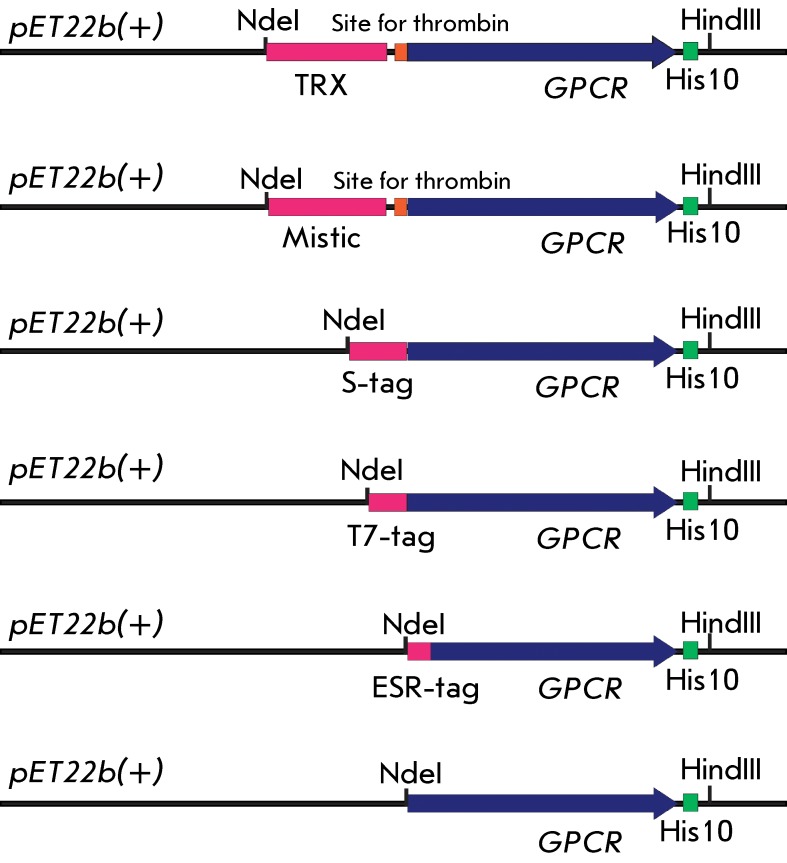
Design of the vectors containing the GPCR genes and additional 5-prime end sequences. *N* -terminal fusion tags coding sequences, GPCR genes, thrombin sites, and polyhistidine sequences are shown in pink, blue, orange, and green, respectively. Restriction endonuclease sites at which the GPCR genes were cloned into the *pET22b(+)* vector are shown

Truncated human β2AR, M1-mAChR, and SSTR5 receptor genes with additional substitutions, and 3-prime end sequences encoding the 10 His residues (His10-tag) (see the Results and Discussion section) are used in this study. The molecular weights of the target proteins were 38.2, 32.6, and 32.7 kDa, respectively. Nucleotide sequences encoding the T7-tag (11 a.a., MASMTGGQQMG), S-tag (16 a.a., MKETAAAKFERQHMDS), TRX (11.8 kDa), and Mistic protein (12.8 kDa) were introduced in one reading frame to the 5-prime end of the truncated GPCR genes ( *Fig. 1* ) using conventional genetic engineering techniques. The nucleotide sequence encoding the ESR-tag (6 a.a., MEEVNL) was introduced to the 5-prime end of the truncated *GPCR* genes to replace the regions encoding N-terminal extracellular fragments of the receptors (see the Results and Discussion section) via single-stage PCR. All these gene constructs were cloned in the pET22b(+) vector (Novagen, USA) under the control of the T7 promoter. The resulting vectors were named *рЕТ22b(+)/GPCR, рЕТ22b(+)/T7-tag-GPCR, pET22b(+)/S-tag-GPCR, рЕТ22b(+)/TRX-GPCR, рЕТ22b(+)/Mistic-GPCR,* and * рЕТ22b(+)/ESR-GPCR (Fig. 1).*

**Cell-free production of GPCR **

GPCRs were synthesized in the continuous cell-free system based on the *E. coli* S30 extract using protocols [15, 21]. The final concentrations of the components of the reaction mixture were as follows: 100 mM HEPES-KOH (Fluka, USA), pH 8.0; 8 mM Mg(OAc) _2_ , 90 mM KOAc, 20 mM potassium acetyl phosphate (Sigma, USA), 20 mM potassium phosphoenolpyruvate (Aldrich, USA), 1.3 mM of each amino acid, except for Arg, Cys, Met, Trp, Asp, Glu, whose concentrations were 2.3 mM; 0.15 mg/ml folic acid (Sigma), each of four ribonucleoside triphosphates at a concentration of 1 mM; proteinase inhibitor (X1 Complete protease inhibitor ^®^ , Roche Diagnostics, Germany); 0.05% of NaN _3_ ; 2% of polyethylene glycol 8000 (Sigma); 0.3 U/µl of ribonuclease inhibitor RiboLock (Fermentas, Lithuania); 0.04 mg/ml of pyruvate kinase (Fermentas, Lithuania); 5.5 µg/ml of T7 polymerase; 0.3 mg/ml of plasmid DNA, 0.5 mg/ml of total tRNA (from *E. coli* MRE 600) (Roche Diagnostics, Switzerland), 30% of the total volume of the reaction mixture of the *E. coli* S30 extract. The feeding mixture (FM) had the same composition, except for the high-molecular-weight components: S30 extract, plasmid, enzymes, and ribonuclease inhibitor. The synthesis was carried out without the addition of any membrane-mimicking media in RM and FM. The RM and FM volumes were 50 and 750 µl, respectively. The RM was placed into the reactor separated from the FM solution with a dialysis membrane (pore size 12 kDa, Sigma, USA), followed by incubation for 20 h at 30 ^o^ C under moderate stirring. 

**Isolation and purification of GPCR samples **

The RMs containing synthesized GPCRs were centrifuged for 15 min at 14000 rpm. The resulting precipitates were solubilized in buffer A (20 mM Tris-HCl, 250 mM NaCl, 1 mM NaN _3_ , pH 8.0) containing 1% of sodium dodecyl sulfate (SDS), 1 mM dithiothreitol, and 8 M urea. The solubilized proteins were transferred to the column with Ni ^2+^ -sepharose (GE Healthcare, Sweden), washed with 10 column volumes of buffer A containing 1% SDS, and eluted with 3 volumes of buffer A containing 1% SDS and 500 mM imidazole. The GPCR samples were dialyzed against buffer A containing 1% SDS. 

The eluate fractions were analyzed by SDS-PAGE and Western blotting using mouse monoclonal antibodies against the hexahistidine sequence (His-tag ^®^ Monoclonal antibody, Novagen, USA). The amount of purified GPCR samples was determined spectrophotometrically at room temperature based on absorption at 280 nm. The CD spectra were recorded at room temperature on a J-810 spectrometer (Jasco, Japan). 

## RESULTS AND DISCUSSION 

**Design of the **

*GPCR* **genes**

The truncated variants of the receptors containing additional point substitutions were used to increase the stability of the GPCR samples and to reduce the aggregation tendency of the proteins. Genetic engineering methods were used to excise the N- and C-terminal extramembrane regions that do not participate in ligand binding [7–9, 22–24]. The deletion of the C-terminal regions of the receptors resulted in the removal of cysteine residues (241,435, and 320), which are presumably the sites of post-translational binding of palmitic acid residues in human β2AR, M1-mAChR and SSTR5 molecules, respectively [7, 23, 24]. In addition, the fragment of the third cytoplasmic loop (L3), which also does not participate in ligand binding, was deleted from the M1-mAChR molecule [8, 9, 25]. The genes obtained encoded the regions 25–340, 19–224/354–426, and 37–319 of human receptors β2AR, M1-mAChR, and SSTR5, respectively. Additional His _10_ -tag sequences were inserted at the 3-prime end of the genes in order to provide further purification of recombinant proteins by Ni ^2+^ affinity chromatography. 

The truncated genes of the β2AR, M1-mAChR, and SSTR5 receptors encoded 10, 9, and 10 cysteine residues, respectively. Among those, only the residues from the extracellular region presumably participate in the formation of disulfide bonds (Cys106–Cys191 and Cys184–Cys190 in β2AR; Cys98–Cys178 and Cys391–Cys394 in M1-mAChR; Cys112–Cys186 in SSTR5, the numeration is provided for the native sequence of the receptors). In order to reduce the aggregation of recombinant proteins due to the formation of “non-native” disulfide intermolecular bonds, transmembrane and cytoplasmatic Cys residues were substituted via site-directed mutagenesis. Thus, the data [26, 27] were used to substitute Cys77, Cys116 and Cys125 residues in β2AR for Val; and to substitute Cys285, Cys327, and Cys265 for Ser. In M1-mAChR, the Cys69, Cys205, Cys417, and Cys421 residues were substituted for Ser [28]. In SSTR5, the Cys129, Cys237, and Cys260 residues were substituted for Ser; the Cys169, Cys218, and Cys220 residues were substituted for Val; and Cys51 and Cys298, for Gly. Furthermore, an additional stabilizing Glu122Trp substitution was introduced to the β2AR sequence [29]. 

**Expression of the **

*GPCR* **genes in cell-free system**

The introduction of membrane-mimicking components to the RM allows to synthesize MPs in the soluble and functionally active forms [13–18]. However, most of these additives (e.g., detergent molecules) may reduce the productivity of the system via the partial or complete inhibition of the synthesis of the target protein [14–17]. For this reason, we did not use membrane-mimicking compounds for the synthesis when performing the comparative analysis of the efficiency of expression of the *GPCR* genes with additional 5-prime end regions. It should be mentioned that the target proteins accumulated as a precipitate in the RM. The precipitates were dissolved in a hard detergent (SDS) in the presence of urea and dithiothreitol as a reducing agent. The amount of synthesized proteins was determined spectrophotometrically after the dissolved precipitates had been purified via Ni ^2+^ affinity chromatography. The synthesis of the target proteins was confirmed using monoclonal antibodies against the hexahystidine sequence. 

**Fig. 2:**
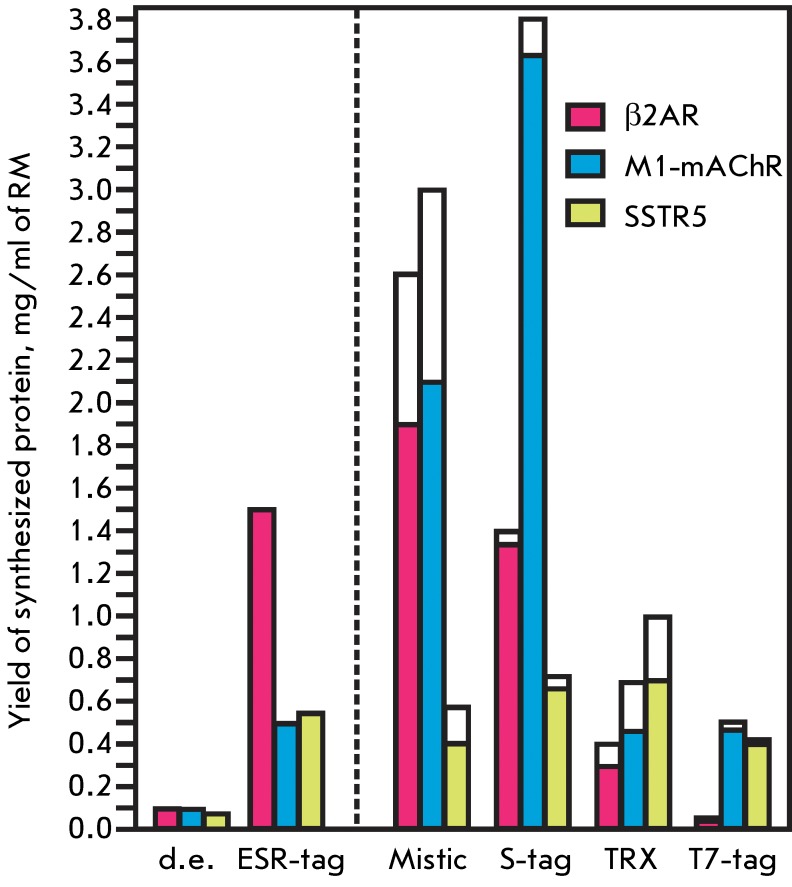
Analysis of the CF synthesis of β2AR, M1-mAChR, and SSTR5 using different *N* -terminal fusion tags. The yields in CF expression of GPCRs without any *N* -terminal tags are designated as “d.e.” (direct expression). Synthesis yields of hybrid proteins are shown by unfilled bars; the yields of the target proteins (colored bars) are shown after the deduction of a part of *N* -terminal partners. Each value represents the average of three experiments. The systematic error does not exceed 15%. The amounts of proteins produced were measured by UV-Vis absorption spectroscopy at 280 nm after purification by Ni ^2+^ -chromatography

As one would expect, the direct expression of the truncated *β2AR* , *M1* - *mAChR* , and *SSTR5* genes in CF systems based on the *E. coli* S30 extract was inefficient. The yield of the target proteins after the purification did not exceed 0.1 mg per 1ml of RM ( *Fig. 2* ). It should be noted that highly efficient production (with a yield of up to 1.6 mg/ml of RM) of bacteriorhodopsin from Gram-positive bacteria *Ex. sibiricum* (ESR) [30], a structural homolog of the GPCRs, which also contains seven TM helices, has been previously observed [15]. We supposed that the low yield of the model GPCRs could be attributed to the low efficiency of translation initiation due to the formation of a secondary mRNA structure at the beginning of the target gene. In order to confirm this assumption, the 5-prime end regions encoding the extracellular N-terminal amino acid residues preceding the first TM helix (25–33, 19–23, and 37–38 in β2AR, M1-mAChR, and SSTR5, respectively) in the truncated GPCR genes were substituted with the nucleotide sequence encoding the first 6 a.a. of bacteriorhodopsin ESR (ESR-tag, the sequence length is indicated with allowance for the N-terminal Met) ( *Fig. 1* ). This substitution allowed one to significantly increase efficiency in the production of the target protein ( *Fig. 2* ). The yield of the ESR-tag-β2AR hybrid protein was comparable to that of the ESR protein, whereas the level of synthesis of the remaining two hybrid proteins (ESR-tag-M1-mAChR and ESR-tag-SSTR5) was approximately three times lower (~0.5 mg/ml of RM). 

**Comparison of the efficiency in GPCR synthesis with various N-terminal fusion tags **

The results obtained have confirmed that the 5-prime end sequence plays a significant role in efficient expression in a cell-free system. However, the yields of the target proteins attained using the ESR-tag presumably were not optimal. Thus, synthesis of recombinant MPs in continuous CF systems based on the *E. coli* S30 extract with yields of up to 4–6 mg/ml of RM has been described in the literature [14]. For further optimization of the synthesis for the model GPCRs, we tested four N-terminal fusion tags. Two of those, the T7-tag (11 a.a.) and TRX protein (11.8 kDa), have previously been used in cell-free production of GPCRs [14, 16, 17], whereas the Mistic protein (12.8 kDa) was used for GPCR production in E. coli [31, 32]. In addition, we tested the sequence encoding the N-terminal fragment (16 a.a.) of ribonuclease A (N-terminal fragment of S-peptide, S-tag), which is used to detect and purify recombinant proteins via affinity chromatography [33], but has never been used as an N-terminal fusion tag for the production of recombinant MPs. In contrast to the method used to design hybrid genes with the 5-prime end fragment encoding the ESR-tag, nucleotide sequences encoding the T7-tag, TRX, Mistic, and S-tag were added in a single reading frame to the 5-prime end of the genes of the truncated GPCR variants ( *Fig. 1* ). 

In most cases, the use of N-terminal fusion tags increased the yield of model receptors, but the yield levels varied for different proteins. Thus, the use of T7-tag increased the yields of M1-mAChR and SSTR5 receptors to ~0.5 mg/ml of RM, whereas the β2AR level stayed low and was comparable to that observed during direct expression. The use of the TRX also provided a small increase in the synthesis of the target proteins to ~0.3–0.7 mg/ml of RM (hereinafter, the amounts of the target proteins are given without the protein-fusion tags part, *Fig. 2* ). Meanwhile, the use of the N-terminal fusion tags Mistic and S-tag allowed one to considerably increase the production of β2AR and M1-mAChR ( *Fig. 2)* . The highest yield of β2AR (~ 1.9 mg/ml of RM) was observed when using the Mistic protein, and the highest yield of M1-mAChR (~ 3.6 mg/ml of RM) was attained for the S-tag hybrid protein ( *Fig. 2* ). However, none of the sequences used has enabled to attain a considerable increase in the SSTR5 yield. The yields of this receptor (0.4–0.7 mg/ml of RM) were very close when using various hybrid constructs ( *Fig. 2* ). It seems that the translation initiation for SSTR5 is not the only crucial factor for providing efficient cell-free synthesis. Further optimization of the nucleotide sequence of the gene (e.g., substitution of the codon variants uncommon for *E. coli* ) is presumably required to increase the production level in a cell-free system. It should be noted that a similar SSTR5 yield (~ 0.5 mg/ml of RM) was earlier observed in the bacterial continuous CF system when using a full-length (nontruncated) hybrid of the receptor with the N-terminal T7-tag sequence [34]. 

**Table:** Free energy of formation of the secondary structure by the 5-prime end mRNA fragment (ΔG, kcal/mol)

Fusion tag/*GPCR*	β2AR	M1-mAChR	SSTR5
Direct expression	-5.6	-8.2	-19.3
ESR-tag	-3.1	-3.5	-6.4
Mistic	-1.3	-1.3	-1.3
S-tag	-3.3	-3.3	-3.3
TRX	-7.8	-7.8	-7.8
T7-tag	-5.5	-7.6	-7.3

Note. The free energy was calculated using the M-fold software [35] for mRNA fragments containing 4 nucleotides upstream of the start codon, the start codon, and 34 nucleotides of the target protein gene or fusion tag, downstream of the start codon

As previously mentioned, the increase in efficiency in protein synthesis when using additional 5-prime end sequences can presumably be attributed to the reduction in the ability of the 5-prime end mRNA fragment to form a secondary structure. To confirm this assumption, the formation of a secondary structure of the 5-prime end mRNA fragment used for GPCR production was modeled. The modeling was performed using the M-fold software to analyze the free energy of formation of the secondary structure of RNA [35]. The free energy of secondary structure formation was calculated for the mRNA fragments containing four nucleotides upstream of the start codon, the start codon, and 34 nucleotides of the target protein gene or the fusion tag downstream of the start codon, as was described in [20]. The computation ( *Table* ) has shown that the native sequences of the truncated receptors can form stable secondary structures (ΔG ~ –5.6, –8.2, and –19.3 kcal/mol for β2AR, M1-mAChR, and SSTR5, respectively). The use of T7-tag and TRX slightly reduces the stability of the secondary structure of the 5-prime end mRNA fragment (ΔG ~ –5.5–7.8 kcal/mol). Meanwhile, the use of the N-terminal sequence of bacteriorhodopsin ESR considerably reduces the stability of the secondary structure of the 5-prime end mRNA fragment in β2AR and M1-mAChR (ΔG ~ –3.1 and –3.5 kcal/mol, respectively). Secondary structures of mRNA characterized by the lowest stability were obtained for Mistic and S-tag sequences (ΔG ~ –1.3 and –3.3, respectively). The qualitative correlation between the calculated energies and the yields of GPCRs indirectly supports the important role of the formation of an 5-prime end mRNA secondary structure in the decrease in the efficiency of translation initiation and, as a consequence, in the total efficiency of the cell-free synthesis. 

Modification of the 5-prime end region of the target protein gene is not the only way to prevent the formation of a secondary mRNA structure and increase efficiency in translation initiation. Nucleotide sequences from the 5-prime end untranslated regions of mRNA can also affect these processes. In this study, we used genetic constructs based on a pET22b(+) vector (Novagene) containing the *lac* -operator sequence inserted between the T7 promoter and the ribosome-binding site (RBS). According to published data, the use of *pIVEX* vectors (Roche Applied Science, USA) lacking the *lac* -operator can increase efficiency in the direct expression of the *GPCR* genes in bacterial CF systems [34]. In order to verify this assumption, we tested efficiency in the direct expression of the truncated *M1-mAChR* gene using the *pIVEX2.3* vector. The yield of the target protein (~ 0.1 mg/ml of RM) in this case was no higher than that obtained via direct expression of the *M1-mAChR* gene cloned in the *pET22b* (+) vector. The data obtained were in close agreement with the results of the investigation of olfactory GPCRs, whose production in a bacterial cell-free system using *pIVEX* vectors was characterized by low efficiency [36]. Moreover, the use of N-terminal fusion tags was also required to provide highly efficient expression of human protein genes cloned into the *pIVEX* vectors [37]. 

**Fig. 3:**
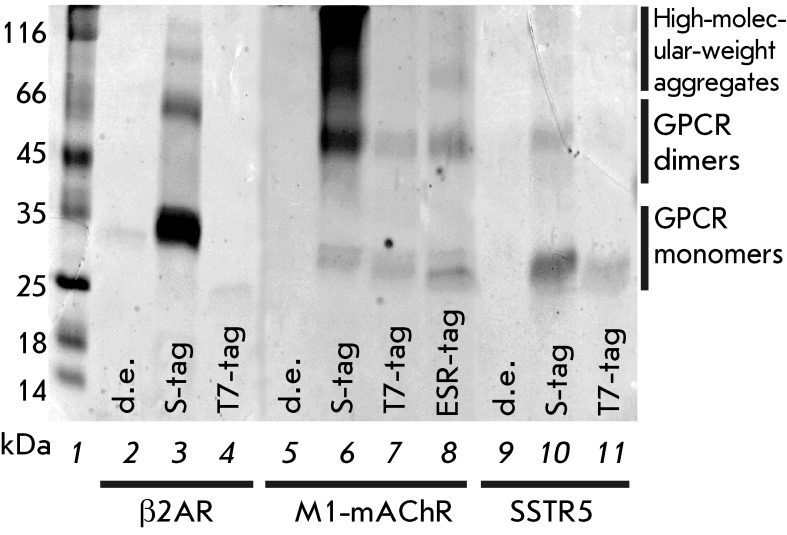
SDS-PAGE analysis of the synthesized GPCRs with different N-terminal fusion tags after purification by Ni ^2+^ -chromatography. Monomers, dimers, trimers and high-molecular-weight aggregates are present in the GPCR samples. 1 – molecular weight markers; 2, 5, 9 – receptors synthesized without N- terminal fusion tags (“d.e.,” direct expression); 3, 6, 10 – receptors with S-tag; 4, 7, 11 – receptors with T7-tag; 8 – ESR-tag-M1-mAChR

**Fig. 4:**
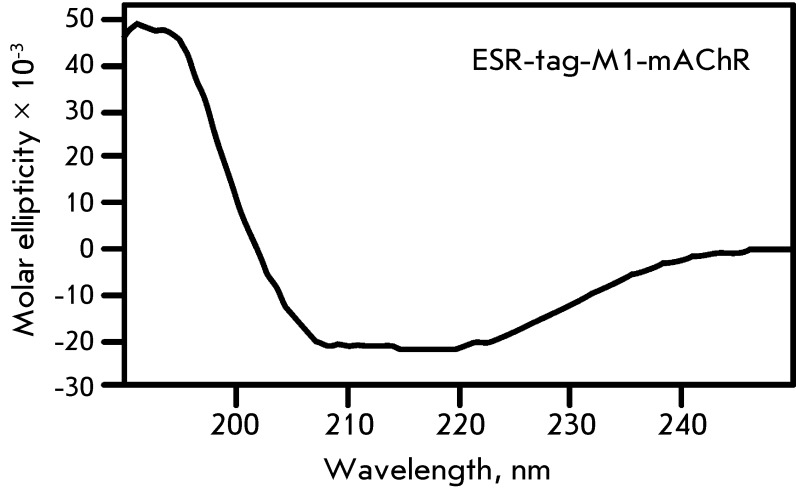
CD spectrum of ESR-tag-M1-mAChR in 1% SDS

Another method to solve the problem of low efficiency in translation initiation in CF systems can include the rational design of the 5-prime end sequence of the target protein gene using synonymous substitutions (without any changes in the encoded amino acid sequence), which is aimed at reducing the mRNA ability to form a secondary structure [20]. This approach was used to produce mammal cytokines when the presence of the fusion tag sequence (N-terminal fragment of cloramphenicol aminotransferase, 5a.a.) hindered the formation of the spatial structure [38]. 

**Analysis of recombinant GPCRs **

Purified GPCR samples solubilized in a hard SDS detergent (1%) were analyzed by SDS-PAGE. The representative gels are shown in *Fig. 3* . The resulting samples, as well as the other MP samples, possess an anomalous electrophoretic mobility, which is presumably caused by incomplete denaturation of MP molecules in SDS [39]. Separate bands corresponding to receptor monomers, dimers, trimers, and higher order aggregates were detected on the gels ( *Fig. 3* ). This behavior is typical for GPCRs, which tend to form dimers and trimers in biological membranes and are prone to spontaneous aggregation due to hydrophobic interactions between TM helices even in hard detergent solutions [31]. The aggregation level of GPCR samples depends on the receptor type, the sequence of N-terminal fusion tags, and presumably on the protein concentration in the sample. Thus, the highest amount of high-molecular-weight aggregates was observed in S-tag-M1-mAChR samples characterized by the most efficient synthesis. The secondary structure of the ESR-tag-M1-mAChR hybrid, which exhibited the lowest degree of aggregation in solution, was analyzed by CD spectroscopy ( *Fig. 4* ). The analysis of the resulting data revealed that the α-helical structure was the predominant one (α-helix – 65%, β-sheet – 4%, β-turn – 9%, and irregular regions – 22%), which attests to the fact that the secondary structure of the receptor is partially formed in the environment of SDS micelles. It should be noted that the content of α-helical elements in a molecule of the truncated M1-mAChR receptor calculated similarly to that in the known crystal structures of M2 and M3-mAChR [8, 9] is supposed to be equal to ~72%. 

Further investigation of recombinant GPCRs requires either an optimization of the procedure of target-protein solubilization from the RM precipitate, followed by the development of renaturation methods for the obtained samples, or the use of membrane-mimicking media during the CF synthesis, which allows to synthesize MPs in the functionally active form in some cases [13,15, 34, 35]. 

## CONCLUSIONS 

The data obtained have demonstrated that the use of amino acid sequences of the ESR-tag, S-tag, and Mistic protein as N-terminal fusion tags allows to achieve a highly efficient production of human GPCRs in a cell-free system based on the *E. coli* S30 extract. Utilization of these sequences provides yields of target protein production (0.6 – 3.8 mg/ml of RM) that are sufficient for further structural and functional studies. The present work is the first to demonstrate the possibility of using the ESR-tag and S-tag to increase the level of heterologous production of MPs. 

## Abbreviations

a.a.: amino acid residue
β2AR: human β2-adrenergic receptor
CF system: cell-free expression system
ESR: bacteriorhodopsin from*Exiguobacterium sibiricum*
ESR-tag: 6 a.a. N-terminal fragment of the ESR
FM: feeding mixture
GPCR: G-protein-coupled receptor
M1-mAChR: human muscarinic acetylcholine receptor M1
MP: membrane protein
RM: reaction mixture
SSTR5: human somatostatin receptor type 5
S-tag: 16 a.a. N-terminal fragment of ribonuclease A
T7-tag: 11 a.a. N-terminal fragment of the leader peptide of the protein 10 of the bacteriophage T7
TM: transmembrane
TRX: thioredoxin from*Escherichia coli*
